# Optimization of Produced Parameters for PA6/PA6GF30 Composite Produced by 3D Printing with Novel Knitting Method

**DOI:** 10.3390/polym17121590

**Published:** 2025-06-06

**Authors:** Selim Hartomacıoğlu, Mustafa Oksuz, Aysun Ekinci, Murat Ates

**Affiliations:** 1 Department of Mechanical Engineering, Faculty of Technology, Marmara University, 34854 Istanbul, Turkey; selimh@marmara.edu.tr; 2 Faculty of Engineering and Architecture, Recep Tayyip Erdoğan University, 53100 Rize, Turkey; 3 Department of Polymer Materials Engineering, Faculty of Engineering, Yalova University, 77200 Yalova, Turkey; 4 Department of Chemistry, Faculty of Science, Niğde Omer Halisdemir University, 51100 Nigde, Turkey; aysun.ekinci@yalova.edu.tr; 5 Department of Materials and Materials Processing Technologies, Yalova Vocational School, Yalova University, 77200 Yalova, Turkey; 6 Department of Chemistry, Faculty of Arts and Sciences, Tekirdag Namik Kemal University, Degirmenalti Campus, 59030 Tekirdag, Turkey; mates@nku.edu.tr; 7 Nanochem Polymer Energy Company, Silahtaraga Mah., University 1st Street, Number: 13/1 Z242/1, 59860 Tekirdag, Turkey

**Keywords:** 3D printer, PA6, glass fiber, composite, Taguchi

## Abstract

The additive manufacturing sector is rapidly developing, providing alternatives for mass production in the polymer composite industry. Due to the direction-dependent mechanical properties and high cost of fiber-reinforced polymeric materials, it is necessary to take advantage of alternative multi-materials and production technologies. In this study, a special geometric-shaped knitting technique was investigated using two different materials. The main material was polyamide 6 (PA6), and the inner or second material was PA6 with a 30 wt.% glass fiber addition by weight (PA6GF30). The special geometric shape, layer thickness, nozzle temperature, and post-heat treatment time were measured as process parameters in the production of the PA6/PA6GF30 composites with the fused deposition modeling (FDM) technique. The Taguchi design method and L9 fractional experiment were used in the experimental study. The mechanical behaviors of the PA6/PA6GF30 samples were obtained using tensile and impact tests. In addition, scanning electron microscopy (SEM) analyses were performed on the fracture lines of the PA6/PA6GF30 samples, and damage analyses were carried out in more detail. The experimental results were sorted using grey relational analysis (GRA). Moreover, the optimal experimental conditions and their related plots were obtained. As a result, the highest tensile strength of the PA6GF30 composite was 89.89 MPa with the addition of a special geometric shape. In addition, the maximum impact resistance value of the PA6/PA6GF30 composite was 83 kJ/m^2^. Hence, the developed knitting method presented many advantages when using the FDM technique, and both were successfully used to produce the PA6/PA6GF30 composites.

## 1. Introduction

In recent years, polymer composites have been among the most innovative materials, especially in the electronic [1,2], aerospace [3], automotive [4], and marine industries [5], due to their superior thermal [6,7], thermomechanical [8,9], and mechanical properties [10,11]. In addition, thermoplastic-based polymer composite materials have been placed on traditional materials that supply significantly reduced-weight materials [12] and recyclability [13]. Different manufacturing methods have been used for thermoplastic-based composite materials, such as injection molding [14], pultrusion [15], and additive manufacturing [16,17]. Additive manufacturing technologies, including fused deposition modeling (FDM) [18,19] and fused filament fabrication (FFF) [20,21], could play an important role in the production of functional materials. FDM technology has recently gained attention as it eliminates the need for a mold [19], reduces post-industrial waste [20], and promotes production sustainability [21,22] in various manufacturing contexts, especially for fiber-reinforced thermoplastic composites. The most common materials for thermoplastic composites include polyamide (PA) [23,24], poly (lactic acid) (PLA) [25,26], poly (ethylene terephthalate-glycol) (PETG) [27,28], and acrylonitrile–butadiene–styrene (ABS) [29]. Polyamide 6 (PA6) has mostly been used in FDM technology for various engineering applications, such as in the automotive, marine, military, and aerospace industries [23,24], due to its mechanical [30], tribological [31], and thermal properties [32]

Generally, PA6’s properties can be improved with carbon fiber, basalt fiber, boron fiber, glass fiber, and natural and inorganic fillers (e.g., talc, calcium carbonate, kaolin, etc.) [33,34]. Peng et al. [35] reported the manufacturing of a lightweight honeycomb short carbon fiber-reinforced PA6 structure using the FDM method with a low cost and an improved mechanical performance. Additionally, Touchard et al. [36] reported the interfacial quality of a continuous carbon fiber-reinforced PA6 composite at the filament/matrix and interlaminar scales.

Sun et al. [37] reported the influences of the carbon fiber content and printing parameters on the micromorphology, thermal properties, and mechanical properties of PA6/CF composites to identify some challenges, such as an inconsistent fiber orientation distribution and void formation during the layer-stacking process. These material challenges could be improved by designing a hybrid material structure rather than producing fully functional components. The tensile strength of the PA6/CF composite material was obtained as 162 MPa, representing a 406% increase when compared to the pure PA6 material. Moreover, the mechanical performance is affected by the FDM processing parameters. Toro et al. [38] reported the effects of 3D-printing processing parameters on the mechanical properties of carbon fiber-reinforced polyamide composites. Morales et al. [39] reported the quasi-static and dynamic crush behaviors of the 3D-printed thin-walled hollow profiles of continuous carbon fiber-reinforced polyamide and continuous glass fiber-reinforced polyamide. Moreover, Beylergil et al. [40] reported the effects of FDM processing parameters, including the raster angle, infill density, extruder temperature, and printing speed, on the Charpy impact strength of CF/PA composites. The results showed that the optimal 3D-printing parameters provided a Charpy impact strength of 10.54 kJ/m², representing an increase of approximately 150%, with the highest contribution coming from the infill density (54.19%) and the lowest from the printing speed (2.84%). Rashid et al. [41] reported the effects of the infill patterns and densities on the deflections (residual stresses and warpage) and mechanical properties of 3D-printed chopped carbon fiber-reinforced PA6 composites. Shashikumar and Sreekanth [42] reported the effects of the raster orientations on the mechanical properties of printed polyamide 6 with 20 wt.% short carbon fibers using the FDM method. The highest tensile strength was 35.20 MPa with a bidirectional ± 45° raster orientation. The highest impact energy was obtained with a raster orientation of 90°. He et al. [43] reported the effect of voids on the tensile and flexural properties of continuous carbon fiber-reinforced PA6 composites fabricated via FDM using a pre-impregnated filament. Additionally, Shashikumar and Sreekanth [42] and Su et al. [44] reported the importance of the fiber orientation and content on the mechanical properties of FDM-printed composites, noting significant improvements in the tensile strength and modulus with carbon fiber reinforcement. Su et al. [44] reported the effects of the fiber content on the thermal properties, fiber length, fiber orientation, tensile properties, porosity, and fracture morphologies of FDM-printed reclaimed carbon fiber-reinforced PA6 composites. The tensile strength and modulus of the PA with 20 wt.% carbon fibers were improved by up to 175% and 329% compared with neat PA, respectively. Hadi et al. [45] reported the effects of temperature and moisture parameters on PA6 and the tensile properties of polyamide reinforced with 20 wt.% glass fiber composites (PA6GF20). Dana et al. [46] reported the influence of the printing parameters, such as the feed rate, building orientations, and layer thickness, on the tensile characteristics of printed CF/PA6. In addition, Sugiyama et al. [47], Xu et al. [48], and Guan et al. [49] reported a composite sandwich structure fabricated using a 3D-printing technique. Xu et al. [48] reported the influence of the process parameters, such as the layer thickness, printing temperature, and printing speed, on the properties of printed continuous glass fiber-reinforced PA6 composites with a sandwich structure using a dual-nozzle 3D-printing technique. The optimal process parameters obtained a flexural strength, flexural modulus, and interlaminar strength of 275.40 MPa, 10.80 GPa, and 26.50 MPa for the printed CGF/PA6 composites with a part thickness of 2.80 mm, respectively.

This study aimed to produce low-cost polymer composites with improved direction-dependent mechanical properties using the 3D-printing technique. A special geometric-shaped 3D knitting technique with two different materials was evaluated in this study. Unlike traditional single-material infill strategies, the proposed knitting method is a novel dual-material internal reinforcement technique, where PA6GF30 is printed within a PA6 matrix using a special geometric pattern via a dual-nozzle FDM system. This approach aims to enhance the mechanical properties directionally by integrating a structurally tailored secondary material during the printing process. The knitting strategy is an infill design that also involves the layer-by-layer embedding of a reinforcement phase (PA6GF30) within the main matrix (PA6), forming a functional internal architecture. This enables better control over interfacial bonding, stress distribution, and mechanical anisotropy, which are critical for performance-driven composite designs.

In particular, 30 wt.% glass fiber-reinforced PA6 (PA6GF30) has been used as a standard engineering material for load-bearing components where enhanced stiffness and dimensional stability are required. Hence, PA6GF30 was selected as the reinforcement material in this study. Pure PA6 was chosen as the main material, while PA6 with a 30 wt.% glass fiber (PA6GF30) addition by weight was chosen as the inner material. The PA6/PA6GF30 composites were produced with a double-nozzle 3D printer, according to the Taguchi experimental design. The effects of the process parameters (the geometric shape dimensions, layer thickness, nozzle temperature, and post-heat treatment time) on the mechanical properties of the PA6/PA6GF30 composite, which was produced by knitting PA6GF30 into pure PA6 with a special cross-section, were investigated. The mechanical properties of the PA6/PA6GF30 composites were examined using tensile and impact tests. Additionally, the morphological analysis of the fracture surfaces of the impact test samples was performed with a scanning electron microscope (SEM). The optimum production parameters were determined by comparing the experimental data and statistical analysis using the GRA method.

## 2. Materials and Methods

### 2.1. Materials

Filaments of pure polyamide 6 (Ultrafuse, PA6) and polyamide 6 reinforced with 30% (by weight) glass fibers (Ultrafuse, PA6GF30) used in the production of the tensile and impact test samples of the PA6/PA6GF30 composites were supplied by BASF SE (Ludwigshafen, Germany). The physical and mechanical properties of the PA6 (at a 245 °C nozzle temperature and in an XY printing direction) and PA6GF30 (at a 250 °C nozzle temperature and in an XY printing direction) filaments are given in Table 1.

### 2.2. Experimental Design

Four factors and three levels of each factor were used in the experimental study. A few parameters that affect tensile strength and impact strength have been used in the literature. Experimental Table L9 was used for the experimental design. While 3^4^ = 81 experiments were performed under normal conditions, nine experiments were carried out using the 1/9 fractional experimental technique. The experimental process parameters are given in Table 2. Moreover, the experimental process parameters, such as the geometric shape dimensions (PA6GF30) (A), layer thickness (B), nozzle temperature (C), and post-heat treatment time (D), were examined.

### 2.3. Design and Production of PA6/PA6GF30 Composites

**Figure 1:**
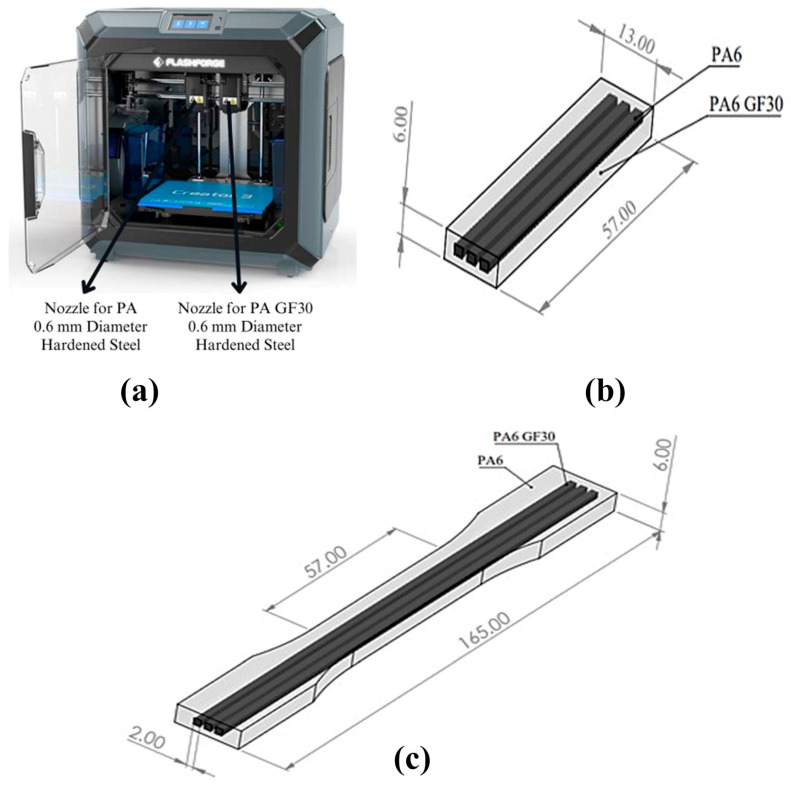
(**a**) FlashForge Creator 3 printer equipped with two 0.6 mm hardened steel nozzles dedicated to PA6 and PA6GF30 extrusion; (**b**) CAD view of the impact test specimen showing internal reinforcement with three PA6GF30 lines embedded in the PA6 matrix (57 × 13 × 6 mm); (**c**) CAD view of the tensile specimen with an extended gauge section (165 mm), also incorporating a PA6/PA6GF30 composite dual-material structure.

**Figure 2:**
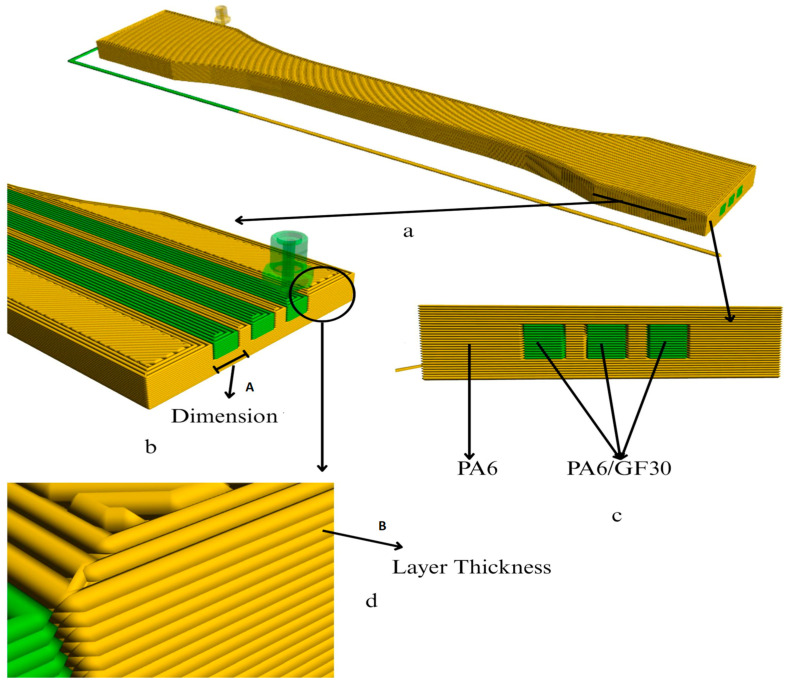
(**a**) Top view showing the specimen’s placement on the 3D printer’s build plate during slicing; (**b**) cross-sectional view along the print path illustrating the geometric dimension (A) of the embedded PA6GF30 reinforcement within the PA6 matrix; (**c**) vertical cross-section perpendicular to the layer deposition direction showing the layered structure and dual-material configuration (yellow: pure PA6; green: PA6GF30); (**d**) detail showing the defined layer thickness (B) between successive deposited lines.

The tensile and impact test specimens of the PA6/PA6GF30 composites were designed according to the ISO standards using the SolidWorks 2020 software. Figure 1 illustrates the novel dual-material knitting strategy used in this study. In this approach, the main body of the composite specimen was printed using pure PA6, while the reinforcement zones—highlighted in the cross-sectional view—were formed by inserting PA6GF30 using a dual-nozzle 3D printer. This configuration enabled the directional control of the mechanical properties by embedding a stiffer secondary material (PA6GF30) into a flexible matrix (PA6). The dimensions, geometries, and cross-sectional views of the tensile and impact test specimens of the PA6/PA6GF30 composites are given in Figure 2. The tensile test and impact test specimens of the PA6/PA6GF30 composites were printed according to the selected parameters based on the Taguchi method using the FDM method, as shown in Table 2. The composite specimens were fabricated using a dual-nozzle FlashForge Creator 3 3D printer (Zhejiang Flashforge 3D Technology Co., Jinhua, China), which offers a build volume of 300 × 250 × 200 mm (X × Y × Z) (Figure 1b). The pad temperature during 3D printing was 90 °C. Heat treatment was applied to the test PA6/PA6GF30 composite samples according to the experimental design in a furnace at 150 °C.

### 2.4. Characterization

The mechanical properties of the PA6/PA6GF30 composites were investigated using tensile and impact tests. The average of three measurements for each sample was calculated. The tensile tests of the PA6/PA6GF30 composites were carried out using a Zwick/Roell (Ulm, Germany) model test device at room temperature according to the ISO 527 standard (Geneva, Switzerland). The impact tests of the unnotched composites were carried out using an Instron 120 D (Norwood, MA, USA) model test device at room temperature according to the ISO 179-1 standard (Geneva, Switzerland).

The absorbed energy was determined for each PA6/PA6GF30 composite at the moment of fracture via an impact test. The total absorbed energy was calculated based on the starting-point and end-point heights of the hammer used according to Equation (1). Afterward, the impact strength was calculated based on the total absorbed energy using Equation (2), considering the cross-sectional area of the PA6/PA6GF30 composite test samples.

*E_T_* represents the total energy (J), *m* denotes the mass, *g* is the standard gravitational acceleration, *h_o_* is the initial height, *h_f_* is the final height, *E_C_* indicates the impact strength [kJ/m^2^], *w* is the sample width, and *t* is the thickness of the samples. Initially, energy losses due to bearing friction and air resistance in the impact-testing machine were measured and included in the calculations.

The morphological behavior of the PA6/PA6GF30 composites was investigated using a JEOL 6610 (JEOL Ltd., Tokyo, Japan) scanning electron microscope (SEM). The SEM analyses were performed on the fracture surfaces of the PA6/PA6GF30 composites after the impact tests. The fracture surfaces of the composite samples were coated with gold (Au) under a vacuum atmosphere before the SEM analysis. An Andonstar digital microscope was used to observe the morphologies of the fracture surfaces of the PA6/PA6GF30 composites after the impact tests.

### 2.5. Statistical Analysis

In this study, the Taguchi method with an L9 orthogonal array was adopted to design experiments involving four process parameters at three levels each. While the Taguchi method efficiently reduced the number of experimental runs from 81 to 9, it did not natively support multi-response optimization. Therefore, grey relational analysis (GRA) was integrated with the L9 design to simultaneously evaluate and optimize three mechanical outputs: tensile strength, impact strength, and Young’s modulus. This combined Taguchi–GRA methodology has been successfully applied in previous composite studies to handle multi-objective decision-making problems.

Six samples of each test condition were produced in this study. Half were used as tensile test samples, and the other half were used as impact test samples. The results of each experiment were determined by averaging the test results. Afterward, the results were analyzed with the Excel software using the grey relational degree and grade values. A factor analysis was performed and interpreted according to the grade values.

The first step of grey relational analysis is to normalize the output variables. In the literature, Essawi et al. used the GRA method in the optimization of 3D-printed carbon fiber-reinforced polyamide composites [50], and the results were successfully optimized. Since the output variables used in this study were the tensile strength and impact strength, the normalization process was carried out using Equation (3). Afterward, the deviation sequence (Δ) was calculated using Equation (4) by subtracting the normalized values from the reference value, 1. In the next stage, the grey relational ranks (*GRCs*) were calculated for each output variable using Equation (5), where *ξ* is the distinguishing coefficient, typically set to 0.5 to balance sensitivity and stability. Using the *GRC* values calculated for each output variable, the grey relational grade (*GRG*) values were calculated using Equation (6). In this study, the weights for the tensile and impact strength values (the output factors) were considered equal and taken as 1.

(1) $$E_{T}=m*g\left(h_{o}-h_{f}\right)$$

(2) $$E_{C}=\frac{E_{T}}{w*t}$$

(3) $$Normalized\;Value=\frac{Absolute\;Value-Minimum\;Recorded\;Value}{Maximum\;Recorded\;Value-Minimum\;Recorded\;Value}$$

(4) Δ=1−Normalized Value

(5) $$GRC=\frac{Minimum\;Delta\;Value+(\xi *Minimum\;Delta\;Value)}{Absolute\;Delta\;Value+(\xi *Minimum\;Delta\;Value)}$$

(6) $$GRG=\frac{1}{\left(Number\;of\;Factors\right)}*\sum_{1}^{Number\;of\;Factors}Factor\;Veight*GRC\;of\;factor$$

## 3. Results and Discussion

### 3.1. GRA Analyses of Mechanical Properties

The mechanical properties, impact strength, tensile strength, and Young’s modulus values of the PA6/PA6GF30 composites are given in Table 3. The GRA method was used in the statistical analysis of the mechanical test results of the composites. The first stage of this method was the normalization process, wherein the data were normalized between zero and one with the approach of largest is best, smallest is best, or target is best. In this study, the higher-is-better approach was preferred for both the impact resistance and tensile stress values. In the second process, the deviation matrix was obtained by calculating the difference from the reference value. For the higher-is-better approach, the reference value was taken as one. The normalization is given in Table 4, and the deviation matrix is given in Table 5. The most important stage of the grey relational analysis method was the calculation of grey relational degrees. At this stage, the grey relational degrees of each output variable (impact strength, tensile strength, and Young’s modulus) were calculated and are given in Table 6.

**Figure 3:**
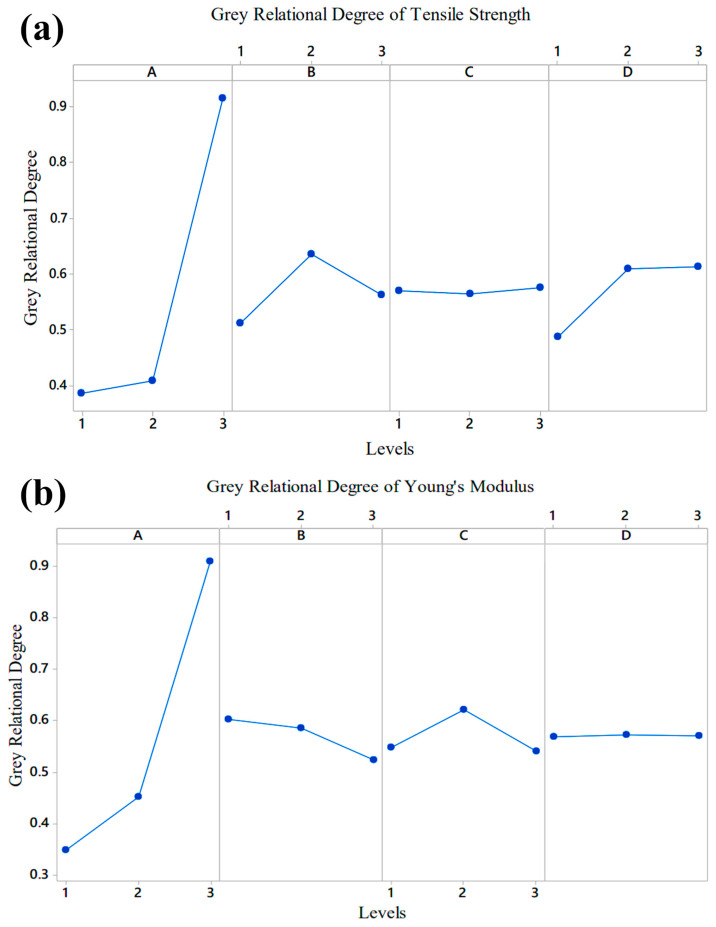
(**a**) The grey relational degrees of the factors for the tensile strength value; (**b**) the grey relational degrees of the factors for Young’s modulus values of the PA6/PA6GF30 composites.

The *GRG* values for each factor and its level were calculated to determine the factor effects. First, plots were drawn for each output variable, and then the overall graph was obtained using the average *GRG* values [50]. The effect plots of the tensile strength of the PA6/PA6GF30 composites are shown in Figure 3a. The graphs show that parameter A, i.e., the geometric shape measure, was the most important input parameter in the experimental design, as it changed to the largest grey relational degree. The most ineffective parameter was the C parameter: that is, the nozzle temperature. The C parameter was low because the nozzle temperature was kept within the range determined by the filament manufacturer. In the experimental study, the optimum production parameters for tensile stress were A1B2C3D2.

In the second stage, the grey relational degrees of the levels of each factor were calculated for Young’s modulus values of the PA6/PA6GF30 composites. The graph shows that the most effective factor in terms of Young’s modulus was A (i.e., the geometric shape measure), and the others were C, B, and D, respectively. The grey relational degrees for Young’s modulus values are given in Figure 3b. The optimal production parameters for Young’s modulus were A3B1C2D1. Figure 3b shows that Young’s modulus increased with the increase in the size of the geometric structure made of the PA6GF30 material in the pure PA6 material.

**Figure 4:**
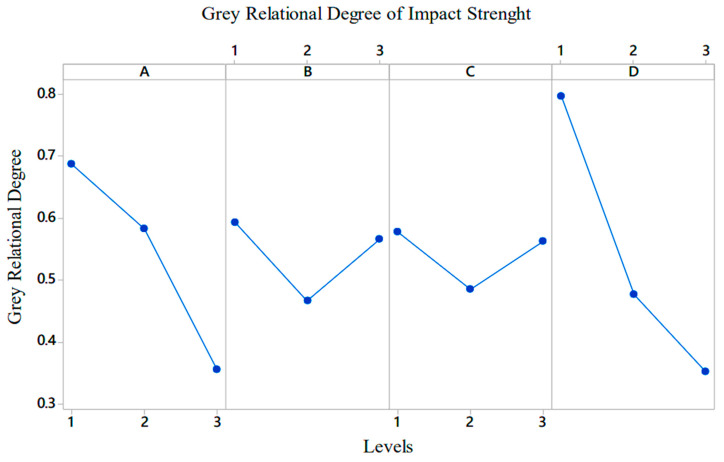
The grey relational degrees of the factors for the impact strength value of the PA6/PA6GF30 composites.

In the third stage, the grey relational degrees of the levels of each factor were calculated for the impact strength value of the PA6/PA6GF30 composites. The graph shows that the most effective factor in terms of impact resistance was D (i.e., heat treatment) and the second most effective factor was A (i.e., geometric shape measurement). The effects of factors B and C were similar. The optimal parameters in terms of the impact resistance were A1B1C1D1. As such, increasing the size of the geometric structure with the PA6GF30 material placed in pure PA6 reduced the impact resistance. The grey relational degrees of the factors for the impact value are given in Figure 4.

**Figure 5:**
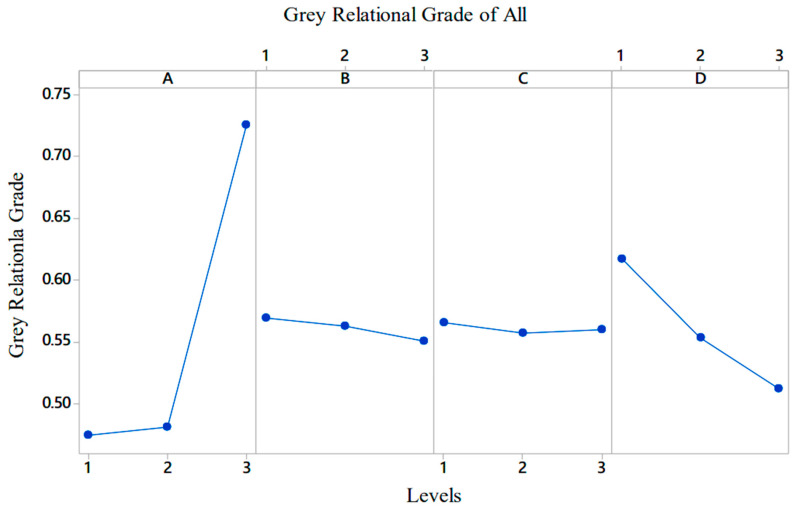
All *GRG* values of the PA6/PA6GF30 composites.

In this study, both tensile strength and impact strength were selected as performance metrics. If evaluated separately, these outputs would yield different optimal conditions. However, when GRA was applied, the normalized values for each output were combined into a single *GRG*, which reflected the overall performance considering all the selected outputs equally. In the last stage, the values were calculated based on all output values according to the general *GRG* value. Here, the weights of each output value were considered equal. The output values could be weighed according to the area where the material combination was used. The *GRG* values were calculated according to the factor levels and are plotted in Figure 5, which shows that factor A was the most effective factor. Factor D came second, factor C came third, and factor B came fourth. When the factors and their levels were evaluated for the optimal selection of the production parameters, it was seen that the A3B1C1D1 or C factor could be A3B1C2D1 or A3B1C3D1 due to its low effect value. Therefore, the A3B1C2D1 parameter combination represented the optimal condition when the tensile and impact strengths were considered together, not just the one with the highest individual grey relational degree (GRD) for a single output.

**Figure 6:**
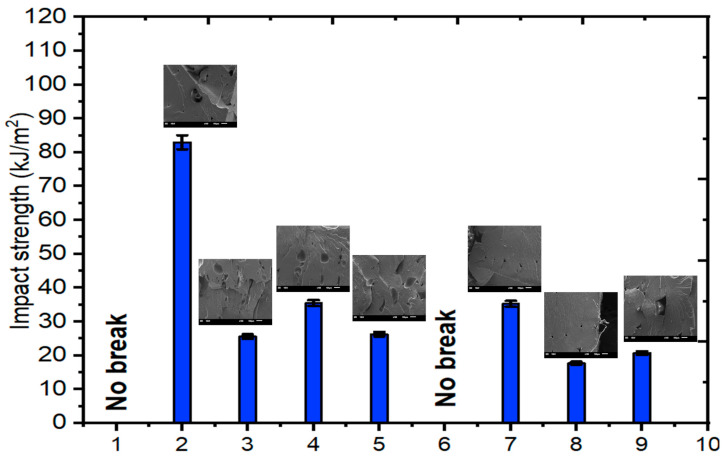
The impact test results and morphological relationship of the PA6/PA6GF30 composites.

**Figure 7:**
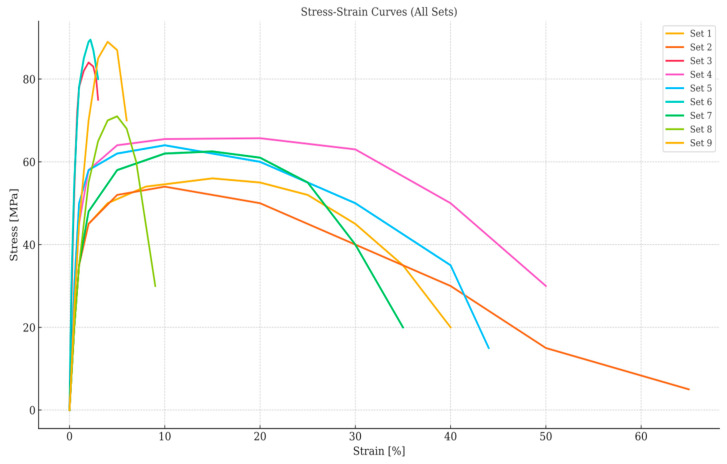
The stress–strain curves of the PA6/PA6GF30 composites.

The impact strength test results and SEM images of the comparison of the PA6/PA6GF30 composites are given in Figure 6. In addition, the tensile stress–strain curves of the PA6/PA6GF30 composites are given in Figure 7. According to the pure PA6 filament’s datasheet, the impact strength value of the sample was 28 kJ/m^2^, the tensile strength was 61.40 MPa, and Young’s modulus value was 2419 MPa. In the experimental sequence, the seventh experiment met these conditions, and the optimal values were obtained from the experiments. The mechanical properties of the PA6/PA6GF30 composites produced under these conditions comprised an impact strength value of 35.27 kJ/m^2^, a tensile strength of 84.98 MPa, and Young’s modulus value of 5082 MPa. Essawi et al. [50] reported that in a 3D-printed continuous carbon fiber-reinforced nylon composite, the optimum values for the filler density, fiber angle, and carbon fiber layer position were expected to have the highest overall ratio and, therefore, the best overall value instead of the maximum value for all parameters. As a result, increasing the dimensions of the special geometric PA6GF30 structure was the most effective parameter change for increasing the tensile strength and Young’s modulus value. The experimental test results show that the mechanical properties of the pure PA6 and PA6GF30 samples in the tensile force direction were improved in the PA6/PA6GF30 composite design. 

### 3.2. SEM Analysis Results

**Figure 8:**
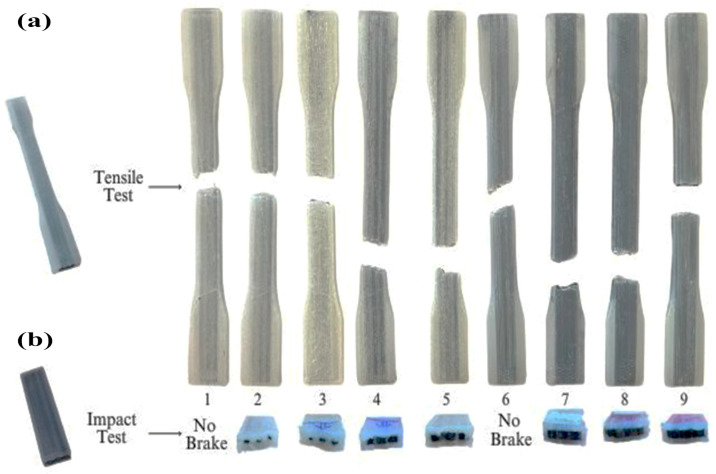
(**a**) The tensile test fractured samples of the PA6/PA6GF30 composites; (**b**) the damage situations occurring in samples due to the impact testing of the PA6/PA6GF30 composites.

**Figure 9:**
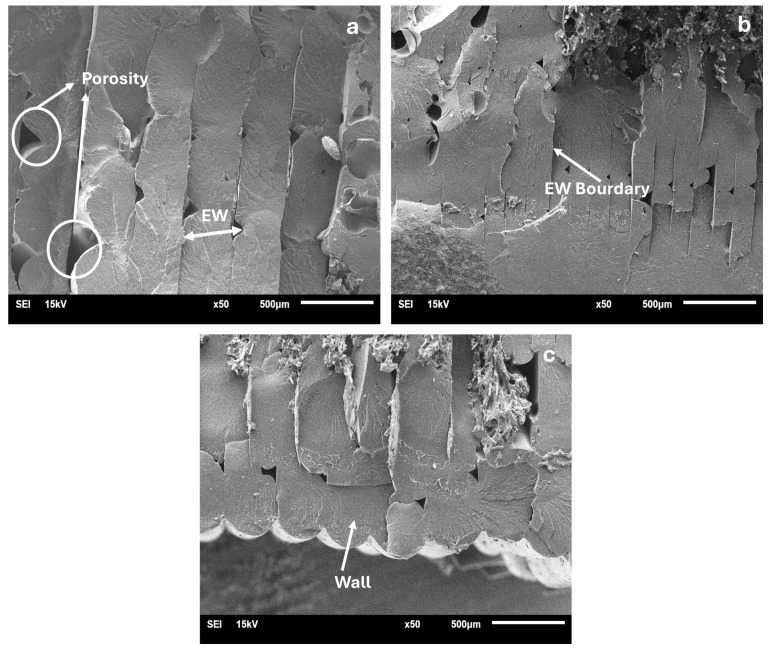
SEM micrographs of the layered structure of the pure PA6 portion of the PA6/PA6GF30 composites: (**a**) number 3; (**b**) number 4; and (**c**) number 9.

**Figure 10:**
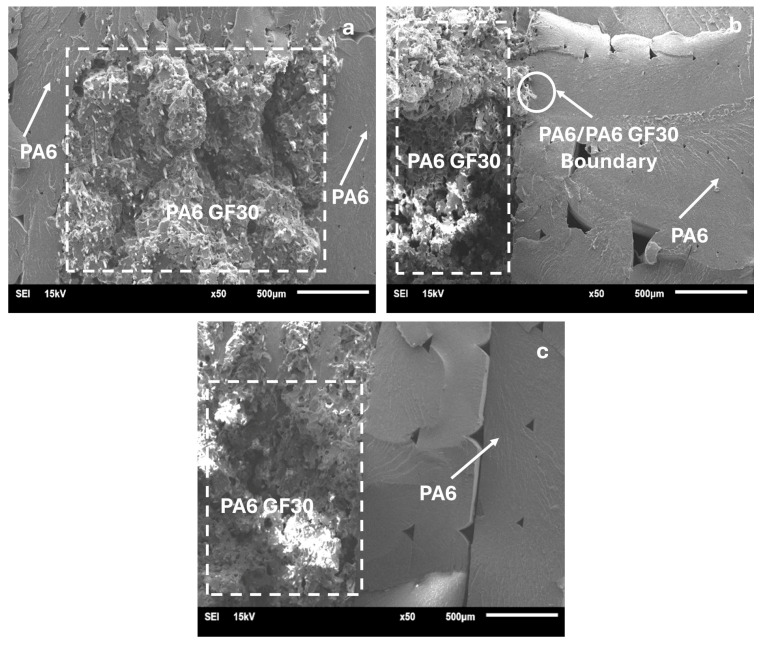
SEM micrographs of the cross-sections of PA6GF30 and the pure PA6 part of the PA6/PA6GF30 composites: (**a**) number 4; (**b**) number 7; and (**c**) number 9.

**Figure 11:**
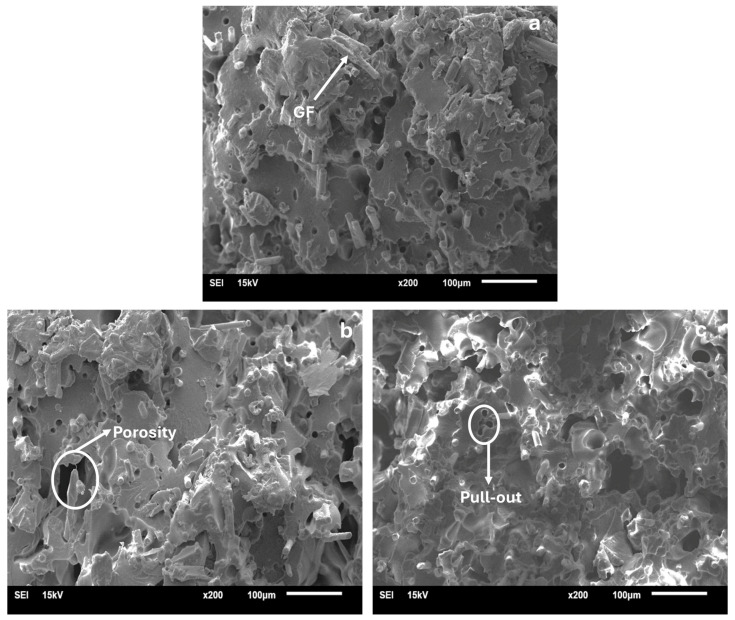
SEM micrographs of the PA6GF30 portion of the PA6/PA6GF30 composites: (**a**) number 7; (**b**) number 8; and (**c**) number 9.

The effects of the process parameters on the morphological structures of the PA6/PA6GF30 composites produced using pure PA6 and PA6GF30 filaments in 3D printing were examined using SEM analysis. In addition, in the first stage, macrophotographs of the fracture behavior of the PA6/PA6GF30 samples were examined in both the tensile test and impact test samples, and the fracture behavior was evaluated. The image of the macrofracture behavior is given in Figure 8. SEM micrographs of the fractured surfaces of the PA6/PA6GF30 composite samples in the impact test are given in Figure 9, Figure 10 and Figure 11. Since samples 1 and 6 of the PA6/PA6GF30 composites did not break during the impact test, SEM images could not be taken. In Hartomacioglu et al.’s study [51], damage analysis was performed for a carbon fiber-reinforced PA6 material. In the study, detailed explanations of the extrusion width (EW), porosity, wall areas, and regions formed in the structure were given within the scope of the damage analysis. A similar methodology was applied in this study, and damage analysis was performed on the SEM images. 

The special square-shaped PA6GF30 material structure within the pure PA6 material showed brittle fracture behavior as the volume increased. Figure 9 displays the layered structure of the pure PA6 part of the selected PA6/PA6GF30 composite samples. The PA6/PA6GF30 samples with the same layer thickness (0.35 mm) are given in Figure 9a,c. Layer thickness variability was visible when comparing Figure 9a,c (0.35 mm) with Figure 9b (0.15 mm). The formation of gaps between pure PA6 layers changed with the changes in the process parameters. In Figure 9a,c, the nozzle temperature had an effect on the gaps between the layers. The SEM micrographs show air gaps resulting from the weak bonding between the pure PA6 layers. Large gaps facilitated the deformation of the test pieces, resulting in lower mechanical values. FDM processes utilizing pure thermoplastics may lead to void formation; however, these voids are generally smaller and less detrimental, provided that key printing parameters—such as the extrusion temperature and infill density—are properly optimized. In contrast, the studies by Sun et al. [37] and He et al. [43] showed that voids present in fiber-reinforced composites significantly compromise their mechanical performance, particularly their tensile strength and flexural properties, thereby increasing the likelihood of material failure under mechanical stress. In the case of fiber-reinforced PA6 composites, poor interlayer bonding can lead to delamination or separation between layers, significantly weakening the material. Hsueh et al. [52] observed that good adhesion between the PLA layers was essential with an increase in the nozzle temperature. Moreover, Rivera-López et al. [53] observed that air voids and triangular voids significantly decreased in samples printed at higher temperatures and were associated with an increase in mechanical parameters, with good adhesion between layers. However, it has been reported that a negative air gap occurs if the nozzle temperature is too high. The literature reports similar results with changes in the process parameters [53,54].

The SEM micrographs of the shape dimensions of PA6GF30 and the pure PA6 part of the selected PA6/PA6GF30 composites are presented in Figure 10. The deformation differences between the pure PA6 and PA6GF30 surfaces are visible in the SEM micrographs. The SEM micrographs and impact test results were compatible (Figure 6). The test pieces’ resistance to deformation was reduced due to the PA6GF30 layer woven into the pure PA6. PA6/PA6GF30 composite parallel layers were visible in the cross-sectional image of the composite structure (Figure 8).

The SEM micrographs of the PA6GF30 part of the selected PA6/PA6GF30 composites with the same shape dimension (2.80 mm) are given in Figure 11. The SEM micrographs of the PA6/PA6GF30 composites exhibited information about fiber thinning, fiber breakage, and the separation of broken fiber pieces in the glass fiber reinforcement in the PA6GF30 part knitted into the pure PA6. Yılmaz investigated the effect of the interaction between the reinforcement element and the matrix interface on the thermomechanical and mechanical properties of short carbon fiber-reinforced PA and short glass fiber-reinforced PA composites [55]. The rough matrix surface in the SEM micrographs indicated a ductile fracture with plastic deformation. It has been emphasized that in PA composites, among various factors such as the fiber volume fraction, fiber orientation, fiber material, matrix material, fillers, and additives, the strength of the fiber–matrix interface, often called the interfacial bond, significantly affects the mechanical properties. The presence of voids resulting from fiber separation from the matrix in the fracture surface analysis was supported in the SEM micrographs, as shown in Figure 11c.

## 4. Conclusions

In this study, the effects of process parameters on mechanical properties were investigated by knitting PA6GF30 special geometric shapes using pure PA6. As this study proposed a novel multi-material printing technique, pure PA6 and PA6GF30—commonly used materials in industrial applications—were selected for the initial investigation. The size and dimensions of the shape, layer thickness, nozzle temperature, and post-heat treatment time were selected as factors. Three levels were determined for each factor. The factor analyses and impact values were determined according to these values. A3B1C2D1 was determined as the optimal process parameter. The tensile strength value of pure PA6, which was 61.40 MPa, was increased to the highest value of 89.89 MPa due to the specially designed shape of the PA6/PA6GF30 composite. This value was obtained at the largest special geometric shape (2.80 mm) cross-section value. In terms of the impact resistance, the impact value for the unnotched Izod test for the pure PA6 material was 28 kJ/m^2^, and the highest value was 83 kJ/m^2^, except for the samples that did not break in the impact test. This value was obtained in the smallest special sample. While the Young’s modulus was 2419 MPa for pure PA6, the highest value was 5082 MPa in the specially shaped structure. These results show that the mechanical properties were significantly improved when specially shaped braids with the PA6GF30 material were formed into the main material, suitable for engineering applications, such as those in the defense and automotive industries, among others. The size of the geometric structure and other production parameters improved the mechanical properties satisfactorily. This technique could be developed and improved with different knitting patterns, orientations, and geometric structures in future studies.

## Figures and Tables

**Table 1:** The material properties of the PA6 and PA6GF30 filaments.

Material Properties	Unit	PA6	PA6GF30
Density	kg/m^3^	1115	1356
Glass transition temperature	°C	49	67
Melting temperature	°C	197	209
Tensile strength	MPa	61.40	78.30
Elongation at break	%	9.60	2.20
Young’s modulus	MPa	2419	5036
Impact strength Izod, notched	kJ/m^2^	5.80	9.20
Impact strength Izod, unnotched	kJ/m^2^	28	38.40

**Table 2:** The process parameters of the PA6/PA6GF30 composites according to the Taguchi experimental method.

Sample Number	Parameters			
	(A)	(B)	(C)	(D)
	Dimension of Geometric Shape (mm)	Layer Thickness (mm)	Nozzle Temperature (°C)	Post-Heat Treatment Time (min.)
1	1.20	0.15	260	0
2	1.20	0.25	270	60
3	1.20	0.35	280	120
4	2.00	0.15	260	60
5	2.00	0.25	270	120
6	2.00	0.35	280	0
7	2.80	0.15	270	0
8	2.80	0.25	280	60
9	2.80	0.35	260	120

**Table 3:** The results of the tensile and impact tests of the PA6/PA6GF30 composites.

Sample Number	Impact Strength (kJ/m^2^)	Tensile Strength (MPa)	Young’s Modulus (MPa)
1	No break	55.88 ± 8.60	1663 ± 531
2	83.00	65.61 ± 0.26	1995 ± 63
3	25.63	62.48 ± 3.95	2035 ± 196
4	35.46	64.13 ± 4.29	3177 ± 130
5	26.28	71.09 ± 1.66	3445 ± 150
6	No break	54.42 ± 3.06	2206 ± 170
7	35.27	84.98 ± 4.36	5082 ± 113
8	17.75	89.89 ± 3.70	4866 ± 213
9	20.73	89.11 ± 1.47	4759 ± 202

**Table 4:** The normalization matrix (higher-is-better).

Sample Number	Impact Strength	Tensile Strength	Young’s Modulus
1	1.0000	0.0412	0.0000
2	0.7933	0.3155	0.0971
3	0.0958	0.2272	0.1088
4	0.2153	0.2738	0.4428
5	0.1037	0.4700	0.5212
6	1.0000	0.0000	0.1588
7	0.2130	0.8616	1.0000
8	0.0000	1.0000	0.9368
9	0.0362	0.9780	0.9055

**Table 5:** The deviation matrix (reference value: 1).

Sample Number	Impact Strength	Tensile Strength	Young’s Modulus
1	0.0000	0.9588	1.0000
2	0.2067	0.6845	0.9029
3	0.9042	0.7728	0.8912
4	0.7847	0.7262	0.5572
5	0.8963	0.5300	0.4788
6	0.0000	1.0000	0.8412
7	0.7870	0.1384	0.0000
8	1.0000	0.0000	0.0632
9	0.9638	0.0220	0.0945

**Table 6:** Input relational degree and grade values of impact strength, tensile strength, and Young’s modulus values.

Grey Relational Degree			Results	
Impact Strength	Tensile Strength	Young’s Modulus	Grey Relational Grade	Ranking
1.0000	0.3427	0.3333	0.5587	5
0.7075	0.4221	0.3564	0.4953	6
0.3561	0.3928	0.3594	0.3694	9
0.3892	0.4077	0.4730	0.4233	8
0.3581	0.4854	0.5108	0.4514	7
1.0000	0.3333	0.3728	0.5687	4
0.3885	0.7832	1.0000	0.7239	2
0.3333	1.0000	0.8878	0.7404	1
0.3416	0.9579	0.8411	0.7135	3

## Data Availability

The original contributions presented in this study are included in the article. Further inquiries can be directed to the corresponding author.
